# Does frequent residential mobility in early years affect the uptake and timeliness of routine immunisations? An anonymised cohort study

**DOI:** 10.1016/j.vaccine.2016.02.049

**Published:** 2016-04-04

**Authors:** Hayley A. Hutchings, Annette Evans, Peter Barnes, Melanie A. Healy, Michelle James-Ellison, Ronan A. Lyons, Alison Maddocks, Shantini Paranjothy, Sarah E. Rodgers, Frank Dunstan

**Affiliations:** a Patient and Population Health and Informatics (PPHI), Swansea University Medical School, Swansea University, Singleton Park, Swansea SA2 8PP, UK; b Cochrane Institute of Primary Care and Public Health, Cardiff University, Heath Park, Cardiff, UK; c Abertawe Bromorgannwg University Health Board (ABM UHB), Singleton Park, Swansea, UK

**Keywords:** Residential mobility, Children, Cohort, Immunisation status

## Abstract

**Background:**

There are conflicting findings regarding the impact of residential mobility on immunisation status. Our aim was to determine whether there was any association between residential mobility and take up of immunisations and whether they were delayed in administration.

**Methods:**

We carried out a cohort analysis of children born in Wales, UK. Uptake and time of immunisation were collected electronically. We defined frequent movers as those who had moved: 2 or more times in the period prior to the final scheduled on-time date (4 months) for 5 in 1 vaccinations; and 3 or more times in the period prior to the final scheduled on-time date (12 months) for MMR, pneumococcal and meningitis C vaccinations. We defined immunisations due at 2–4 months delayed if they had not been given by age 1; and those due at 12–13 months as delayed if they had not been given by age 2.

**Results:**

Uptake rates of routine immunisations and whether they were given within the specified timeframe were high for both groups. There was no increased risk (odds ratios (95% confidence intervals) between frequent movers compared to non-movers for the uptake of: primary MMR 1.08 (0.88–1.32); booster Meningitis C 1.65 (0.93–2.92); booster pneumococcal 1.60 (0.59–4.31); primary 5 in 1 1.28 (0.92–1.78); and timeliness: primary MMR 0.92 (0.79–1.07); booster Meningitis C 1.26 (0.77–2.07); booster pneumococcal 1.69 (0.23–12.14); and primary 5 in 1 1.04 (0.88–1.23).

**Discussion:**

Findings suggest that children who move home frequently are not adversely affected in terms of the uptake of immunisations and whether they were given within a specified timeframe. Both were high and may reflect proactive behaviour in the primary healthcare setting to meet Government coverage rates for immunisation.

## 1 Introduction

The overall aim of routine childhood immunisation is to protect all children against preventable childhood infections [1], [2]. Primary and booster vaccines are offered to all children in the UK as part of a routine schedule to protect against diphtheria, tetanus, pertussis, polio, haemophilus B influenza, measles, mumps, rubella, meningitis C and pneumococcal infection amongst other things.

Previous research has shown that children who experience frequent childhood house moves have poorer physical and mental health both in child and adulthood, compared with children who are more residentially stable [3], [4], [5], [6]. There have however been conflicting findings regarding the effect that frequent relocation has on immunisation uptake. Data from the Millennium Cohort Study found that children who lived in families which had moved during pregnancy or more than two times after the birth of the child were more likely to be partially immunised with the primary immunisations and unimmunised against measles, mumps and rubella. Residential mobility was not associated with not being unimmunised with the primary vaccines, or for the single MMR vaccine use [7]. The authors suggested that the decision not to immunise represents an active decision not to do so by the parents, whilst partial immunisation may be more representative of difficulties in getting vaccinations due to higher mobility.

Studies of highly mobile communities in China and India indicate that the immunisation coverage of mobile children was significantly less than of non-mobile children [8], [9]. Work in the UK in travelling communities has similarly documented reduced level of uptake of immunisations in this mobile community [10]. However, researchers in Finland have reported that there was no association between address changes and use of primary care services [11].

‘Looked after children’ (defined in the U.K. as being within the care of the Local Authority Government) have been shown to be more likely to have an incomplete immunisation record, and where it is complete, to have received immunisations later than those children living in their own homes [12], [13], [14]. Parental lifestyle, including moving home, has been suggested as a possible reason for non-immunisation [13]. A child exposed to frequent residential relocation, even if they are not ‘looked after’, may have similar un-met health needs.

We carried out an analysis of record linked data on a large anonymised population based cohort of children born in Wales between 1 January 1990 and 31 December 2008 to compare the rates for routine immunisations, and whether they were up to date in children who moved home frequently within the first year of life compared to children who did not move.

## 2 Methods

### 2.1 Data sources

This study used data from the Wales Electronic Cohort for Children (WECC) held within the SAIL databank [15]. SAIL is part of the national research infrastructure in Wales based at Swansea University, UK and is a relational database capable of linking anonymized data at individual and household level across many health and health-related data sets [16]. Phase one of the WECC consists of linked anonymized records for over 800,000 children born or living within Wales between 1 January 1990 and 31 December 2008. The individual-level anonymized data on these children that were used for this study were obtained from numerous sources: the Welsh Demographic Service (WDS), a continually updated record of children living in Wales; community child health records from the National Community and Child Health Database (NCCHD); and births and deaths from the Office for National Statistics (ONS).

The information on individuals is linked together from different datasets using Anonymised Linking Fields (ALFs) and information at the household level can be linked together using a similarly constructed Residential Anonymised Linking Field (RALF) [17], [18]. RALFs are assigned to each child based on their current address using the Welsh Demographic Service (WDS) dataset held within SAIL. This is compiled from address changes provided by patients to their General Practitioner. Some short term residential moves however may not be registered as they rely on the patient notifying their General Practitioner of an address change. This unique set-up enables longitudinal analyses to be undertaken on data for groups of individuals living together in the same household, including the ability to follow movement between residences over time [19].

### 2.2 Cohort development and composition

We collated data on immunisation status electronically from the NCCHD. Children were included in the cohort if they had a week of birth between 01/01/1990 and 31/12/2008, were born in Wales, had an exact match to an NHS number, had a live status at birth and for whom immunisation data were available. We excluded stillbirths, infant deaths and children moving into or out of Wales from our analysis.

### 2.3 Measure of exposure

For the purposes of this study, a residential move was defined as ‘a change of residence that was registered with a General Practitioner’. The number of house moves was calculated using data from the Welsh Demographic Survey (WDS) linked to the RALFs. In this analysis we calculated the number of residential moves from the week of birth until the age the immunisation was due. For the primary 5 in 1 vaccine we calculated residential moves up to age 4 months; for primary MMR, booster Meningitis C and booster pneumococcal vaccines we calculated residential moves up to age 12 months.

### 2.4 Measurement of outcome

The outcome measures of interest were uptake of routine immunisations and whether they were given by an agreed timeframe up to age two. We pre-defined whether immunisations were delayed in being given for each immunisation of the standard UK schedule in consultation with a group of community paediatricians and according to previous published literature [12] (see Table 1).

## 3 Statistical analysis

We analysed the data using SPSS version 19. We compared children who had not moved prior to the relevant immunisation on time date, to children who had moved frequently and compared uptake rate of immunisations and whether they were delayed or not. We defined frequent movers as those who had moved: 2 or more times in the period prior to the final scheduled on-time date (4 months) for 5 in 1 vaccinations (against diphtheria, tetanus, pertussis, polio and haemophilus B); and 3 or more times in the period prior to the final scheduled on-time date (12–13 months) for primary MMR, booster pneumococcal and booster Meningitis C vaccinations. Using the child's week of birth and week of immunisation we were able to determine whether immunisations were received or not for each child and whether they were delayed or not in being given. We compared data for uptake of immunisations and whether they were given by a specified timeframe between the non-moving and frequent moving groups. We calculated univariate odds ratios (95% confidence intervals) to determine if there was any difference between the non-movers and the frequent movers.

## 4 Ethical approval

National Research Ethics Service (NRES) guidance does not require ethical review for anonymised databank studies. We obtained approval from the independent Information Governance Review Panel (IGRP), whose membership includes Caldicott Guardians and other Information Governance professionals, lay people and representatives from the National Research Ethics Service (NRES) to use SAIL to answer the specific house moves research question [15], [16].

## 5 Results

After accounting for dates for the roll-out programme for a particular immunisation (see Table 1), the final cohort sizes for analyses were: primary MMR 512,018; primary 5 in 1 vaccine 464,756; booster Meningitis C 207,916 and booster pneumococcal 11,428.

Table 2 shows the percentage of children who received the primary MMR, booster Meningitis C, booster pneumococcal and Primary 5 in 1 immunisations and whether there was any delay evident with increasing frequency of house moves. The coverage of the immunisation programme was high across all categories: MMR (>93%), meningitis C (>97%), pneumococcal (>96%) and 5 in 1 (>95%). The proportion of children who received their immunisations by the specified timeframes was also high across all house moves categories: MMR (>86%), meningitis C (>97%), pneumococcal (>96%) and 5 in 1 (>79%).

Table 3 shows the comparison of immunisation uptake between the non-moving and frequently moving groups. Moving home frequently did not increase the odds of not being immunised when compared to not moving home. Table 4 shows the comparison of the primary immunisations and whether they were delayed in being given between the non-moving and frequently moving groups. Again the odds of immunisations being given by the specified timeframe were not different between the frequently moving and non-moving groups.

## 6 Discussion

Our study, which examined the association between frequent residential moves on uptake rate and whether immunisations were delayed for the studied routine primary and booster immunisations scheduled in early childhood in the UK, demonstrated that frequent home moves do not appear to have a detrimental effect on immunisation status.

We found no differences between frequent and non-movers in terms of rate of uptake of the primary and booster immunisations studied or whether the administration of these was delayed. The uptake rate of immunisations and whether they were given by the specified timeframe was high across all house moves categories. Findings from the Millennium Cohort Study, suggested that children from families that moved more frequently or during pregnancy were more likely to be partially immunised with primary immunisations and unimmunised against MMR [7]. The authors concede that reasons for non-immunisation of children are likely to be based on parental choice not to immunise, whereas partial immunisation is more likely to be due to practical difficulties related to residential mobility. Although we did not examine the completeness of the full immunisation programme, instead only examining selected parts, our findings do however agree with the findings from the Millennium Cohort Study in relation to primary immunisations, where frequent residential moves did not result in any change in immunisation uptake rate [7].

Research has suggested that reasons for non-immunisation or partial immunisation may be related to parental lifestyle factors which includes frequent residential mobility, family size and lone parent status [13], [20], [21], [22]. Findings from other countries and highly mobile travelling communities have also suggested that the reason mobile children had poor immunisation rates were because of their limited access to health services [8], [9], [10]. Only those children whose parents have registered them with a healthcare provider were included in our cohort. It is likely therefore that our study population still has good access to and utilisation of health services despite moving frequently. The Department of Health has set national targets for immunisation rates with the aim that by the age of two, 95% of children will be immunised against diphtheria, tetanus, polio, pertussis, Hib, measles, mumps and rubella [23]. In addition, general practitioners are now incentivised to reach immunisation targets by the Quality and Outcomes Framework (QOF) [24]. The lack of correlation seen in our study could be due to a number of reasons. The immunisation uptake rate within Wales has been high for a number of years and could demonstrate the robustness of the vaccination program within Wales (http://www.wales.nhs.uk/sites3/page.cfm?orgid=457&pid=54144), thereby making it difficult to detect any differences. It may also be that Wales has differing socio-demographic factors or social support systems when compared to other geographical areas resulting in a decreased association between social stress and residential mobility; or it may be due to our study design. These issues require further exploration in future studies.

We conducted this study using a large retrospective data cohort. There are strengths and limitations with this methodology. One strength of our study was that the size of the cohort with a large sample size for all the outcomes of interest. In addition, many of the outcomes were collected in a standardised way and as such this allowed a more rigorous comparison of the data.

In our study, a residential move was defined as the change of address registered with the General Practitioner. It was recognised that a move may only be next door or to any location within Wales. It was noted that some short term residential moves may not be registered and would therefore not be captured as they relied on the families notifying their health care providers when their address changed. This was recognised as a limitation of the study. The frequency of residential moves in our study is therefore likely to be under reported. In addition, our study did not examine details regarding the distance of the move. For the purposes of our analysis, only the frequency of moves in our pre-specified periods was examined. The timing of the moves was not examined in our models. It is possible that unregistered children may not be detected as not being immunised or under-immunised or delayed. It is also likely that unregistered children are more likely to be under vaccinated due to decreased health seeking behaviour. Findings from the Millennium Cohort Study have indicated that high residential mobility resulted in increased likelihood of non-response to their survey [25]. Although we used different methods and data sources, it is still possible that this had an effect on our findings. Finding alternative ways to identify these children may ameliorate this problem, but would be difficult within the context of this anonymised cohort study.

Our definitions of whether the vaccinations were delayed or not in being given also may not be as sensitive when compared to other previously used definitions of timeliness [26], [27], [28], [29] and were based largely on clinical discussions. This may have resulted in an overestimation of the number of children protected from infectious diseases through immunisation given that even a small delay will make some children vulnerable and could impact on herd immunity in the general population [26]. We recommend further research employing more stringent definitions of timeliness within our cohort to determine whether this would have any impact on our findings.

## Funding source

This project was funded through a number of grants. The Welsh Government New Ideas Social Research Fund supported the initial research. The study was also supported by two UK research centres. The Centre for the Development and Evaluation of Complex Interventions for Public Health Improvement (DECIPHer) is a UKCRC Public Health Research Centre of Excellence. Funding from the British Heart Foundation, Cancer Research UK, Economic and Social Research Council (RES-590-28-0005), Medical Research Council, the Welsh Assembly Government and the Wellcome Trust (WT087640MA), under the auspices of the UK Clinical Research Collaboration, is gratefully acknowledged.

The Centre for the Improvement of Population Health through E-records Research (CIIPHER) is one of four UK e-health Informatics Research Centres within the Farr Institute funded by a joint investment from: Arthritis Research UK, the British Heart Foundation Cancer Research UK, the Chief Scientist Office (Scottish Government Health Directorates), the Economic and Social Research Council, the Engineering and Physical Sciences Research Council, the Medical Research Council, the National Institute for Health Research, the National Institute for Social Care and Health Research (Welsh Government) and the Wellcome Trust (grant reference: MR/K006525/1).  

**Conflict of interest statement**

The authors declare that there is no conflict of interest.

## Figures and Tables

**Table 1:** Routine immunisations with definitions of specified timeframes for timeliness.

Primary immunisation	Scheduled age	Pre-specified timeframe for immunisation	Start of routine electronic data collection
Primary 5 in 1	2–4 months	12 months	1992
Primary MMR	12–13 months	24 months	Pre 1990
Booster pneumococcal	12–13 months	24 months	2006
Booster Meningitis C	12–13 months	24 months	1999

**Table 2:** Routine immunisations received and immunisations by specified timeframe with increasing frequency of moves.

	Frequency of moving house (Age 0 to <12 months old, except 5 in 1^a^)				
	0 (*n*, %)	1 (*n*, %)	2 (*n*, %)	3+ (*n*, %)	Total
Immunisation received					
Primary MMR (due at 12–13M)					
Yes	439,387 (93.2%)	61,969 (94.0%)	8073 (94.3%)	1502 (93.6%)	510,931
No	32,142 (6.8%)	3949 (6.0%)	489 (5.7%)	102 (6.4%)	36,682
Total	471,529 (100%)	65,918 (100%)	8562 (100%)	1604 (100%)	547,613

Booster Meningitis C (due at 12–13M)					
Yes	201,150 (97.5%)	32,009 (98.5%)	4252 (98.1%)	811 (98.4%)	238,222
No	4914 (2.4%)	487 (1.5%)	76 (1.8%)	12 (1.5%)	5489
Total	206,064 (100%)	32,496 (100%)	4328 (100%)	823 (100%)	243,711

Booster pneumococcal (due at 12–13M)					
Yes	35,990 (96.7%)	6499 (97.6%)	1008 (97.5%)	187 (97.9%)	43,684
No	1229 (3.3%)	160 (2.4%)	26 (2.5%)	4 (2.1%)	1419
Total	37,219 (100%)	6659 (100%)	1034 (100%)	191 (100%)	45,103

Primary 5 in 1 (due at 4M)^b^					
Yes	454,802 (95.7%)	16,398 (96.4%)	1124 (96.3%)		472,324
No	19,186 (4.0%)	556 (3.3%)	37 (3.2%)		19,779
Total	473,988	16,954	1161		492,103

Immunisation by specified timeframe					
Primary MMR (1 doses required; by 24M)					
Yes	387,383 (87.7%)	53,411 (87.8%)	6847 (88.0%)	1255 (86.7%)	448,896
No	54,561 (12.3%)	7438 (12.2%)	930 (12.0%)	193 (13.3%)	63,122
Total	441,944 (100%)	60,849 (100%)	7777(100%)	1448 (100%)	512,018

Booster Meningitis C (due by 24M)					
Yes	171,071 (97.0%)	26,787 (97.9%)	3433 (97.6%)	652 (97.6%)	201,943
No	5279 (3.0%)	587 (2.1%)	83 (2.4%)	16 (2.4%)	5965
Total	176,350 (100%)	27,374 (100%)	3516 (100%)	668 (100%)	207,908

Booster pneumococcal (due by 24M)					
Yes	8959 (96.5%)	1777 (97.5%)	265 (96.7%)	46 (97.9%)	11,047
No	325 (3.5%)	46 (2.5%)	9 (3.3%)	1 (2.1%)	381
Total	9284 (100%)	1823 (100%)	274 (100%)	47 (100%)	11,428

Primary 5 in 1 (due by 12M)^b^					
Yes	357,816 (79.9%)	13,060 (83.4%)	865 (83.8%)		371,741
No	90,225 (20.1%)	2596 (16.6%)	167 (16.2%)		92,988
Total	448,041 (100%)	15,656 (100%)	1032 (100%)		464,729

a House moves frequency for Primary 5 in 1 was between age 0 < 4 months for receipt and timeliness of immunisation.

b Frequent house moves classified as 2 or more moves as time prior to immunisation schedule due date only 4 months.

**Table 3:** Record of completion for each routine immunisation in frequently moving (3 or greater moves^a^) and non-moving children.

Immunisation	Frequent movers % complete (number complete/eligible)	Non-movers % complete (number complete/eligible)	Difference (non-movers − frequent movers)	Odds ratio (95% confidence intervals)
Primary MMR	93.6 (1502/1604)	93.2 (439,387/471,529)	−0.4	1.08 (0.88–1.32)
Booster Meningitis C	98.5 (811/823)	97.6 (201,150/206,064)	−0.9	1.65 (0.93–2.92)
Booster Pneumococcal	97.9 (187/191)	96.7 (35,990/37,219)	−1.2	1.60 (0.59–4.31)
Primary 5 in 1	96.8 (1124/1161)	96.0 (454,802/473,988)	−0.8	1.28 (0.92–1.78)

a Frequently moving defined as 2 or more moves up to 4 months for Primary 5 in 1 vaccine.

**Table 4:** Record of immunisations by specified timeframe for each routine immunisation in frequently moving (3 or greater moves^a^) and non-moving children.

Immunisation	Frequent movers % by specified timeframe (number by specified date/eligible)	Non-movers % by specified timeframe (number by specified date/eligible)	Difference (non-movers − frequent movers)	Odds ratio (95% confidence intervals)
Primary MMR	86.7 (1255/1448)	87.7 (387,383/441,944)	1.0	0.92 (0.79–1.07)
Booster Meningitis C	97.6 (652/668)	97.0 (171,071/176,350)	−0.6	1.28 (0.77–2.07)
Booster Pneumococcal	97.9 (46/47)	96.5 (8959/9284)	−1.4	1.69 (0.23–12.14)
Primary 5 in 1	83.8 (865/1032)	80.0 (357,816/448,041)	−3.8	1.04 (0.88–1.23)

a Frequently moving defined as 2 or more moves up to 4 months for Primary 5 in 1 vaccine.
